# Flowthrough pretreatment with very dilute acid provides insights into high lignin contribution to biomass recalcitrance

**DOI:** 10.1186/s13068-016-0660-5

**Published:** 2016-11-10

**Authors:** Samarthya Bhagia, Hongjia Li, Xiadi Gao, Rajeev Kumar, Charles E. Wyman

**Affiliations:** 1 Department of Chemical and Environmental Engineering, Bourns College of Engineering, University of California Riverside, 900 University Ave, Riverside, CA 92521 USA; 2 Center for Environmental Research and Technology, Bourns College of Engineering, University of California Riverside, 1084 Columbia Ave, Riverside, CA 92507 USA; 3 BioEnergy Science Center (BESC), Oak Ridge National Laboratory, PO Box 2008 MS6341, Oak Ridge, TN 37831 USA

**Keywords:** Batch, Dilute acid, Flowthrough, Lignocellulosic biomass, Liquid hot water, Pretreatment, Poplar, Recalcitrance

## Abstract

**Background:**

Flowthrough pretreatment is capable of removing much higher quantities of hemicellulose and lignin from lignocellulosic biomass than batch pretreatment performed at otherwise similar conditions. Comparison of these two pretreatment configurations for sugar yields and lignin removal can provide insights into lignocellulosic biomass deconstruction. Therefore, we applied liquid hot water (LHW) and extremely dilute acid (EDA, 0.05%) flowthrough and batch pretreatments of poplar at two temperatures and the same pretreatment severity for the solids. Composition of solids, sugar mass distribution with pretreatment, sugar yields, and lignin removal from pretreatment and enzymatic hydrolysis were measured.

**Results:**

Flowthrough aqueous pretreatment of poplar showed between 63 and 69% lignin removal at both 140 and 180 °C, while batch pretreatments showed about 20 to 33% lignin removal at similar conditions. Extremely dilute acid slightly enhanced lignin removal from solids with flowthrough pretreatment at both pretreatment temperatures. However, extremely dilute acid batch pretreatment did realize greater than 70% xylan yields largely in the form of monomeric xylose. Close to 100% total sugar yields were measured from LHW and EDA flowthrough pretreatments and one batch EDA pretreatment at 180 °C. The high lignin removal by flowthrough pretreatment enhanced cellulose digestibility compared to batch pretreatment, consistent with lignin being a key contributor to biomass recalcitrance. Furthermore, solids from 180 °C flowthrough pretreatment were much more digestible than solids pretreated at 140 °C despite similar lignin and extensive hemicellulose removal.

**Conclusions:**

Results with flowthrough pretreatment show that about 65–70% of the lignin is solubilized and removed before it can react further to form low solubility lignin rich fragments that deposit on the biomass surface in batch operations and hinder enzyme action. The leftover 30–35% lignin in poplar was a key player in biomass recalcitrance to enzymatic deconstruction and it might be more difficult to dislodge from biomass with lower temperature of pretreatment. These results also point to the possibility that hemicellulose removal is more important as an indicator of lignin disruption than in playing a direct role in reducing biomass recalcitrance.

**Electronic supplementary material:**

The online version of this article (doi:10.1186/s13068-016-0660-5) contains supplementary material, which is available to authorized users.

## Background

Photosynthesis is nature’s way of capturing and storing solar energy in the form of plant biomass. The reaction pumps the sun’s energy uphill to combine carbon dioxide and water that have no heating value to form glucose and other plant components [1]. Plant biomass is an enormous renewable energy resource that can be utilized as a raw material for conversion of the sugars and aromatics that comprise much of biomass into commodities, with fermentation of sugars to ethanol receiving considerable attention for fulfilling future fuel needs. The structural portion of plants contains three major components, cellulose, hemicellulose, and lignin and is referred to as lignocellulosic biomass. Cellulose and hemicellulose can be converted into sugars by enzymes while lignin can be broken down into aromatics. Enzyme driven deconstruction typically requires prior pretreatment to achieve commercially viable product yields, which, *inter alia*, may remove or alter the hemicellulose and lignin in biomass to increase the surface area of polysaccharides accessible to enzymes [2]. Although cellulose may be altered in aqueous pretreatments, most of the original structure remains in the pretreated solids. Very high temperatures or high chemical concentrations are required to thermo-chemically breakdown cellulose into glucose, but increased sugar losses via parasitic degradation pathways result in low glucose yields [3]. However, as noted in an opinion article outlining important needs for advancing cellulosic ethanol “Just as the three most important contributions in real estate are location, location, location, the three most important factors for commodity products are yield, yield, yield” [4]. In addition to high sugar yields, high sugar concentrations in pretreatment and enzymatic hydrolysis stages are desirable to keep downstream costs low. Fortunately, cellulase enzymes are capable of breaking down most of the cellulose in pretreated biomass with little subsequent degradation, thereby achieving the high yields essential to commercial competitiveness. Furthermore, enzymatic hydrolysis is the only known technology that has the potential to convert crystalline cellulose, the largest polymer in lignocellulosic biomass, into glucose at near theoretical yields. The most significant obstacle to commercializing such biological based processes is the cost of enzymes due to the large doses needed to obtain high sugar yields from pretreated biomass [4–6]. Settling for lower yields to reduce enzyme loadings shifts the cost burden to the feedstock and pretreatment. Thus, it is vital to understand and advance pretreatment and enzymatic hydrolysis technologies for biomass deconstruction and their integration to devise new approaches to reduce the amount of enzyme required to achieve high sugar yields.

Numerous mechanical and chemical pretreatment options have been studied and developed over the years. Among the chemical types, pretreatments can be broadly divided into aqueous pretreatments and solvent based pretreatments, with some comparative studies providing more details [7–9]. Aqueous pretreatments such as those with liquid hot water (LHW) and dilute acid have the advantage that they do not use organic solvents and are usually carried out in batch reactors in laboratory studies. Organic solvents, in spite of some pretreatment benefits, can be expensive and raise issues about fire and pressure containment hazards. Moreover, carryover of residual organic solvent to downstream operations may inhibit microbes and/or enzymes [10]. Aqueous batch pretreatments can remove a large portion of hemicellulose, modify but not remove much of the lignin, increase biomass accessible surface area, and increase cellulose digestibility by enzymes [11].

Most laboratory pretreatments are performed in batch operations and commercial continuous pretreatments generally transport the liquid and solids together. However, flowthrough pretreatment can be valuable for deciphering deconstruction mechanisms occurring during aqueous pretreatments, as continuous removal of soluble fractions from biomass provides valuable insights into the sequence in which components are removed and the effect on the features of the remaining solids that can be compared to results for batch pretreatments at otherwise similar conditions. Pioneered by the late Dr. Ortwin Bobleter of the University of Innsbruck, Austria [12–15], the distinguishing feature of flowthrough pretreatment is that both lignin and hemicellulose are removed from biomass during pretreatment, in contrast to batch pretreatments that remove relatively little lignin [16–18]. This difference is believed to result from lignin that is covalently attached to hemicellulose oligomers initially moving into solution with the highly soluble oligomers for both batch and flowthrough pretreatments. It has been further hypothesized that the lignin-hemicellulose oligomers bonds break when held at reaction conditions for batch pretreatments, with the low solubility lignin fragments then condensing onto the solids biomass. On the other hand, flowthrough pretreatment removes these lignin-oligomer fragments before they have time to breakdown to lower solubility products [19]. Other than a lignin-hemicellulose fragmentation mechanism, some studies indicated that lignin depolymerization at acidic conditions can proceed through formation of carbenium intermediates followed by cleavage of β-*O*-4 linkages in lignin [20–22]. As a result of enhanced lignin removal by flowthrough pretreatment, digestion of the glucan left in flowthrough solids by enzymes can achieve near theoretical glucan yields [23]. Furthermore, flowthrough pretreatment has shown that flow rate and solids concentration enhance xylan solubilization beyond that predicted by models that only account for acid concentration, temperature, and time in accordance with Saeman’s and other first order kinetic models [17, 24–27].

Even though flowthrough pretreatment produces solids that are more digestible by enzymes than solids from batch pretreatment, the large amount of water needed dilutes the sugar stream and leads to high costs for downstream sugar concentration or fermentation product recovery. In addition, the high heat capacity of water results in excessive energy consumption or high capital costs for effective heat exchange. Flowthrough operations are also more complex than batch. In spite of these challenges, there has been interest in scaling up flowthrough pretreatment from bench scale to pilot scale. Recently, Kilpeläinen et al. [28] successfully applied hot water flowthrough pretreatment to spruce and birch at a 300 L scale and discussed the impact of channeling in the biomass bed, an important topic in continuous pretreatment system design. In addition, partial flowthrough systems are applied commercially for wood chip pretreatment [29]. Interest in flowthrough systems is also growing for extracting compounds such as flavonoids, terpenes, phytosterols, tannins and protein from biomass. Extraction of the small amounts of active medicinal ingredients from herbal plants can be particularly desirable, with extractions in room temperature water or solvents important to avoid degrading medicinal qualities [30]. Flowthrough pretreatment may enhance extraction yields and throughput by improving mass transfer rates, while the limited time in solution could allow higher temperature operation that reduce manufacturing costs. The high value of such intermediates and nutraceuticals could potentially compensate for the challenges in downstream processing of dilute solutions.

Most flowthrough pretreatments were carried out with just hot water at high temperatures (LHW). Although such operations provide interesting insights, the deconstruction patterns observed may not be applicable to lower temperatures that would be more desirable for commercial pretreatments. For example, lower temperature pretreatments would lower energy costs, and resulting lower pressures would reduce wear and tear on solid biomass feeding systems and vessel wall thickness and associated capital costs [31, 32]. This study, therefore, focused on investigating deconstruction of plant biomass for flowthrough pretreatment at lower temperatures and severities than applied in the past to understand factors that could account for less effective pretreatments at more commercially relevant conditions when applied to poplar, a fast growing hardwood and model research plant focused on by the Bioenergy Science Center (BESC), Oak Ridge, TN. This comparative study is unique in its examination of sugar yields and lignin removal for application of two pretreatment configurations, aqueous pretreatment with extremely dilute acid and pretreatment with just hot water at lower severities. In addition, a pretreatment temperature of 140 °C was applied that is lower than most studies on flowthrough pretreatment. While other studies varied the pretreatment severity, this study held the liquid hot water pretreatment severity as well as dilute acid combined severity constant for two temperatures 140 and 180 °C, allowing for comparison of the effects of temperature. As per our knowledge, this is the first report of enzymatic hydrolysis sugar yields from extremely dilute acid batch and flowthrough pretreated solids.

## Methods

Bioenergy Science Center standard poplar (BESC STD) was provided by National Renewable Energy Laboratory (NREL), Golden, CO as chips and knife milled through a 1 mm screen (Model 4 Wiley Mill Thomas Scientific, Swedesboro, NJ) at University of California at Riverside. Milling of chips in this size range was chosen for comparison with prior studies [17, 33] as large particle sizes or chips may increase mass transfer resistance and impact yields [34]. The moisture content of BESC STD was 7.3% as determined by a halogen moisture analyzer (Model HB43-S, Mettler Toledo, Columbus, OH).

The reaction conditions for batch and flowthrough pretreatments are summarized in Table 1. Batch pretreatments were carried out at a 2% poplar solids loading in a 1L Parr^®^ reactor (Parr Instruments, Moline, IL, USA) equipped with dual pitch-blade turbine type impellers turning at 200 rpm to mix a total mass of 700 g. 2% solid loading was chosen instead of the usual 5 or 10% loading in laboratory studies because of the limited availability of material.

**Table 1:** Process conditions

Identification number	Aqueous condition	Type	Severity factor^a^	Temperature (°C)	Time (min)
1	Liquid hot water	Flowthrough	log_10_R_0_ = 3.4	140	192
2		Flowthrough		180	12
3		Batch		140	192
4		Batch		180	12
5	Extremely dilute acid (0.05%)	Flowthrough	logCS = 1.4	140	192
6		Flowthrough		180	12
7		Batch		140	192
8		Batch		180	12

^a^R_0_ = t*exp((*T*−100)/14.75)), where *t* is the time in minutes and *T* is the temperature in °C. log_10_R_0_ is called the pretreatment severity factor. logCS = log_10_R_0_ − pH is called the combined severity factor

Extremely dilute acid (EDA) pretreatments were carried out with 0.05 wt% of sulfuric acid based on liquid mass, i.e., 686 g, with biomass moisture content included in the liquid mass. Milled biomass was used as-is (no Soxhlet extraction) and soaked overnight to ensure good moisture penetration into the poplar before pretreatment. The Parr^®^ reactor was rapidly heated in a fluidized sand bath (Model# SBL-2D, 4 kW, Techne Corp., Princeton, NJ) set at 360 °C with the temperature maintained at target values as measured by a K-type thermocouple (Omega Engineering Co., Stamford, CT) inserted in the reactor by adjusting the level of the reactor vessel in contact with sand. For both batch and flowthrough pretreatments, time zero for pretreatment was when the temperature was within 2 °C of the required pretreatment temperature. The variation in pretreatment temperature was less than 1 °C. When the chosen pretreatment time was reached, the vessel was quickly removed from the sand bath and quenched in a large water tank at 10 °C [27, 35]. It took 2 and 3 min to heat-up the batch and flowthrough reactors to 138 and 178 °C, respectively, and took around the same time to cool them down to 40 °C after reaction at the desired pretreatment time was over.

Because the flowthrough pretreatment system used in this study was described previously [23], only key points will be outlined here. The 1/2″ ID by 6″ long reactors were loaded with 1 g of BESC STD on a dry basis, and an extremely dilute acid (0.05%) solution (EDA) or deionized water (LHW) was continuously fed to the flowthrough reactor. Although batch dilute acid pretreatments are typically performed in 0.5–1.0 wt% percent sulfuric acid, it was necessary to limit the acid concentration to 0.05 wt% because higher acid concentrations would rapidly corrode our Type 316 stainless steel flowthrough reactor system at the temperatures run. This limitation proved fortunate as side-by-side comparisons of results from batch and flowthrough pretreatment with liquid hot water (LHW) and extremely dilute acid (EDA) at moderate pretreatment severity provided unanticipated insights into biomass deconstruction. A 20 mL/min flow rate applied to the flowthrough reactor with an internal volume of 19.2 mL resulted in a linear velocity of 15.8 cm/min and a liquid residence time of 0.93 min. The temperatures listed in Table 1 for flowthrough reactions were maintained by setting the fluidized sand bath temperature 9 °C higher than the target value. A back pressure gauge controlled the reactor pressures at 40.6 and 160 psig for 140 and 180 °C, respectively.

Following pretreatment, the liquid was removed from the solids by filtration with Whatman^®^ glass microfiber filter and thoroughly washed with deionized water until neutral pH was achieved, to be sure no acid, soluble sugars, or other solubilized products were left in the pretreated solids. For compositional analysis, the wet solids were dried at 37 °C for several days until the moisture content dropped to about 4–7%. This moisture content was accounted for in the dry weight calculation. Biomass composition and determination of oligomeric sugars were determined according to standard NREL procedures “Determination of Structural Carbohydrates and Lignin in Biomass” [36] and “Determination of Sugars, Byproducts, and Degradation Products in Liquid Fraction Process Samples” [37], respectively. All sugar analyses were carried out on a Waters^®^ e2695 Separations Module equipped with a Waters^®^ 2414 RI detector and a Biorad^®^ Aminex^®^ HPX-87H column conditioned with a 5 mM sulfuric acid mobile phase at 65 °C. Untreated and washed pretreated solids were enzymatically hydrolyzed for 120 h with 100 mg of Accellerase^®^ 1500 cellulase protein/g glucan in the unpretreated biomass. Enzymatic hydrolysis of never-dried solids was performed according to standard NREL procedure, “Enzymatic Saccharification of Lignocellulosic Biomass” [38]. Accellerase^®^ 1500 enzyme that had an 82 mg/mL protein content as determined by the standard BCA method [39] was generously provided by DuPont Industrial Biosciences, Palo Alto, CA. UV-vis spectrophotometry of acid soluble lignin was carried out by a Spectramax^®^ M2e Plate Reader (Molecular Devices, Sunnyvale, CA) equipped with SoftMax^®^ Pro data acquisition software in a Costar^®^ UV 96 well-plate with absorbance of water blank taken into account for correction of sample absorbance. Three replicates of each sample were kept in a 96 well-plate for measurements. A 25 L/(g cm) absorption coefficient and 240 nm wavelength were employed in line with the standard NREL procedure [36] to calculate acid soluble poplar lignin concentrations by the Beer Lambert Bouguer Law. Three replicates were kept for each sample for all standard biomass procedures. Graphs and statistical analyses were carried out using OriginPro^®^ v. 2015 (OriginLab Corp., Northampton, MA) statistics and graphing software.

### Calculations

The mass of each sugar was converted to the mass of its corresponding anhydrous form by multiplying glucose values by 0.9 and xylose measurements by 0.88 to account for the mass of water added to each during hydrolysis. Enzymatic hydrolysis loading was based on mg of protein per gram glucan in the unpretreated biomass to allow fair comparison of the effect of overall enzyme loadings on performance for each pretreatment. Mass units were in grams, volumes in liters, and concentrations in grams per liter. Stage 1 and Stage 2 refer to the pretreatment and enzymatic hydrolysis stages, respectively.

1. $${\text{Mass of polymeric sugar in solids before pretreatment}}\, = \,{\text{Dry weight of biomass fed to pretreatment reactor }}*{\text{ fraction of polymeric sugar in unpretreated biomass}}$$
2. $${\text{Mass of total liquid before pretreatment }} = {\text{ Mass of water added to the reactor }} + \, \left( {{\text{fraction moisture }}*{\text{ wet weight of biomass added to the reactor}}} \right) \, + {\text{ mass of acid }}\left( {\text{if added}} \right)$$
3. $${\text{Solid yield }}\% \, = \,\frac{{\begin{array}{*{20}c} {\text{Wet weight of biomass solids recovered from pretreatment reactor*}} \\ {\left( {1 - {\text{moisture content fraction}}} \right) *\,100} \\ \end{array} }}{\text{Dry weight of unpretreated biomass fed to reactor }}$$
4. $${\text{Mass of liquid after pretreatment }} = {\text{ Mass of total liquid before pretreatment }} + \, \left[ {\left( { 1- {\text{ solid yield fraction}}} \right)\,*\,{\text{dry weight of biomass fed to pretreatment reactor}}} \right]$$
5. $${\text{Volume of liquid after pretreatment }} = \,\frac{{{\text{Mass of liquid after pretreatment}}\,({\text{g}})}}{{{\text{Measured density of liquid after pretreatment}}\,\left( {\frac{\text{g}}{\text{L}}} \right)}}$$
6. $${\text{Stage 1 sugar yield}}\,\% \, = \,\frac{{\begin{array}{*{20}c} {{\text{Concentration of monomeric sugar from HPLC}}\, *\,} \\ {{\text{Volume of liquid after pretreatment}}\, *\,{\text{Anhydrous correction factor}}\, *\, 1 0 0} \\ \end{array} }}{\text{Mass of polymeric sugar in solid before pretreatment}}$$
7. $${\text{Lignin or xylan removal}}\,\% = \,\frac{{\begin{array}{*{20}c} {\left[ {{\text{dry weight of biomass fed to pretreatment}}\, *\,{\text{lignin or xylan fraction of unpretreated biomass}}} \right] - } \\ { [ {\text{dry weight of pretreated solids from the reactor}}\, *\,{\text{lignin of xylan fraction of pretreated solids]}}} \\ \end{array} }}{{\left[ {\text{dry weight of biomass fed to pretreatment*lignin or xylan fraction of unpretreated biomass}} \right]}}$$
8. $${\text{Enzyme loading }} = \,\frac{\text{mg of protein per gram glucan in enzymatic hydrolysis flask}}{{({\text{mass of glucan in solids after pretreatment}}/{\text{mass of glucan in solids before pretreatment}})}}$$
9. $${\text{Enzymatic saccharification efficiency}}\,\% = \,\frac{{\begin{array}{*{20}c} {{\text{Concentration of monomeric sugar from HPLC}}\, *\,{\text{anhydrous correction factor}}\, *\,} \\ {{\text{total reaction volume of enzymatic hydrolysis flask}}\, *\, 1 0 0} \\ \end{array} }}{\text{mass of glucan or xylan in enzymatic hydrolysis flask}}$$
10. $${\text{Stage 2 sugar yield}}\,\% \, = \,\frac{{{\text{Enzymatic saccharification efficiency fraction}}\, *\,{\text{mass of glucan or xylan fed to stage }}\,2\, *\,100}}{\text{Mass of polymeric sugar in solid before pretreatment}}$$
11. $${\text{Total Stage 1}} + 2 {\text{ glucan or xylan yield}}\,\% \, = {\text{ Stage 1 yield}}\,\% \, + {\text{ Stage 2 yield}}\,\%$$
12. $$ {\text{Total Stage 1}} + 2 \left( \text{glucan } + \text{ xylan} \right)\text{ yield}\,\% \, = \text{ Stage 1 glucan } + \text{ xylan yield}\,\% \, + \text{ Stage 2 glucan } + \text{ xylan yield}\,\% $$

## Results and discussion

### Solids compositions and yields

**Fig. 1:**
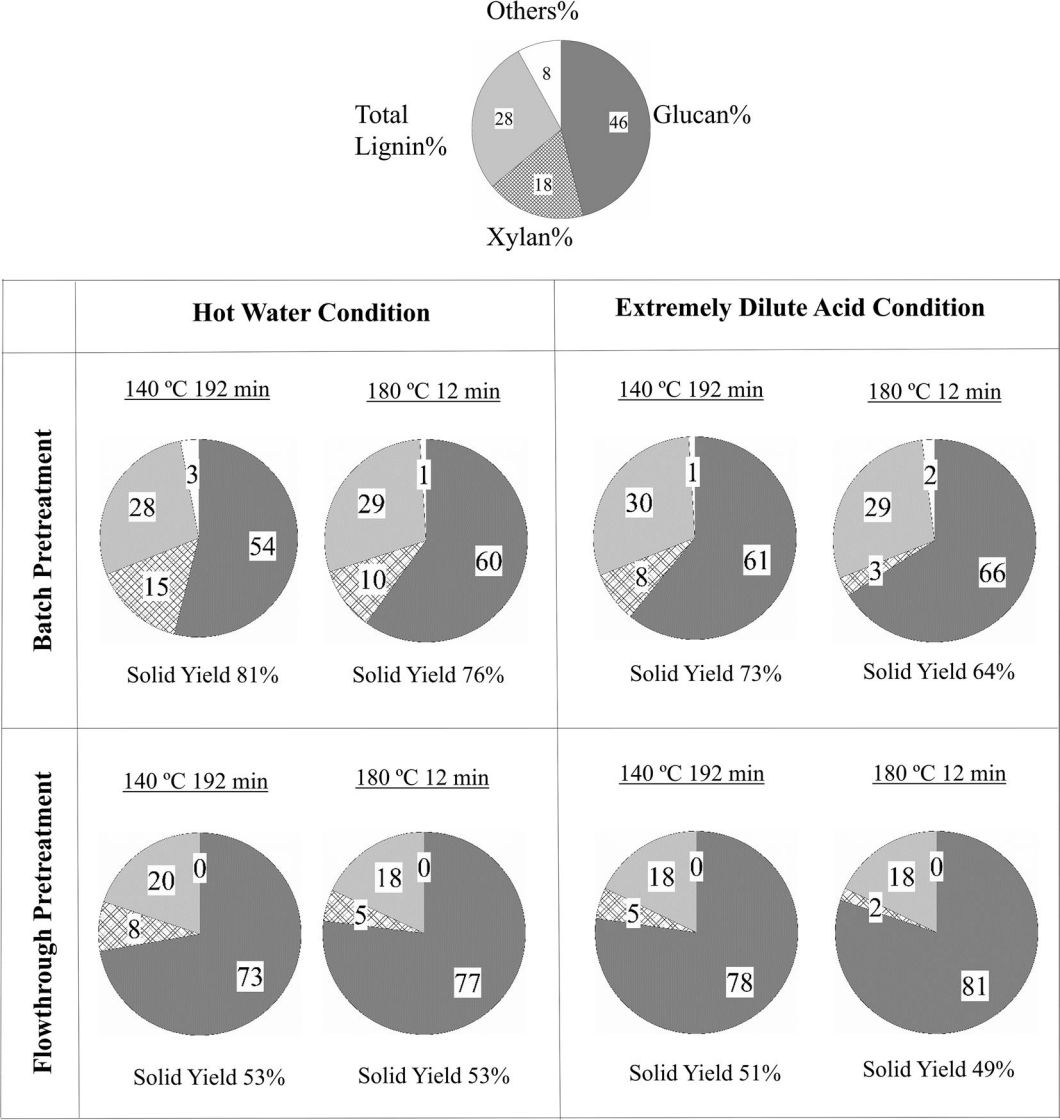
Composition and solid yields. Composition of poplar prior to pretreatment and yields and compositions of solids produced by liquid hot water (LHW) and extremely dilute acid (EDA) batch and flowthrough pretreatments. Number labels have been rounded-off to the nearest whole number for clarity. The components other than glucan, xylan, and total lignin labeled “Others” were not measured experimentally

Figure 1 shows glucan, xylan and total lignin (acid insoluble lignin plus acid soluble lignin) contents in unpretreated and pretreated solids, with each chart accompanied by the percent of the original mass recovered as solids after pretreatment (solid yield%). Most of the glucan is contained in cellulose, the core component of lignocellulosic biomass, and a small amount as xyloglucan in the primary cell wall of hardwoods like poplar [40]. Most of the hemicellulose in poplar is composed of xylose. Total lignin in this figure is the sum of acid insoluble lignin (AIL) (also known as Klason lignin or K-lignin) and acid soluble lignin (ASL). It can be seen on going from left to right in Fig. 1 that solid yields dropped with increasing temperature for both LHW and EDA batch pretreatments. In addition, solid yields dropped by 5% points for higher temperature batch LHW pretreatment and 9% points for higher temperature batch EDA pretreatment. Furthermore, flowthrough pretreatments displayed different trends in solid yields than batch pretreatments in that those from batch pretreatments dropped with increasing temperature and with addition of acid while solid yields from LHW flowthrough pretreatments at both 140 and 180 °C remained at 53% and only changed from 51 to 49% when the temperature was increased from 140 to 180 °C for EDA flowthrough pretreatments. In all situations, about half of the hardwood biomass was solubilized at these moderate severities.

Because increasing flow rate and not just temperature was previously shown to reduce solid yields [17, 27], it is important to note that the flow rate of 20 mL/min applied here to a 0.5” by 6” reactor resulted in a linear velocity of 15.8 cm/min and liquid residence time of 0.93 min. By comparison, when Mok and Antal [18] applied a flow rate of 1 mL/min at a lower linear velocity of around 6 cm/min and somewhat greater liquid residence time of 1.26 min in a 0.46 cm by 7.6 cm reactor, biomass solubilization values were between 40 and 60% with LHW flowthrough pretreatment at 230 °C after 2 min for 10 biomass species including a solid yield of around 50% for poplar. LHW flowthrough pretreatment of corn stover at 200 °C for 12 to 20 min by Liu and Wyman [27] resulted in a solid yield between approximately 50–55% at a linear velocity of 10.7 cm/min and residence time of 1.68 min. On the other hand, when Bobleter and co-workers [12] applied flow rates between 11 and 14 mL/min to a larger 560 ml reactor vessel containing aspen hardwood at 180 °C, the resulting residence time of between 40 and 50 min produced a 57% solids yield after 30 min. Another article reported solid yields between 48 and 58% from pretreatment of poplar at temperatures of 180 and 200 °C for residence times of 2 and 0.67 min through application of 3 and 9 mL flow rates in a 6 mL flowthrough reactor [41, 42]. After 20 min in the Liu and Wyman study [27], 45% of the corn stover was solubilized at 200 °C. Thus, these results from several independent studies show that flowthrough pretreatment from 140 to 230 °C at LHW severity factors [43] between 3.4 and 4.1 solubilizes roughly half of the biomass. Furthermore, this result does change significantly at much lower severities due to limited hemicellulose and lignin solubilization, or at very high severities that trigger cellulose degradation. Our preliminary work in applying a flow rate of 20 mL/min at 140 °C for just 12 min, the latter two conditions corresponding to a low severity factor log_10_R_0_ of 2.2, gave a 90% solid yield in LHW versus 78% in EDA higher temperatures (data not shown). Cellulose solubilization is known to increase drastically at temperatures above 240 °C and can reach 100% at a severity factor 5.5 [44].

The high removal of xylan and lignin in these pretreatments translated into enhanced glucan content in the pretreated solids, as shown in Fig. 1. Furthermore, the glucan content for solids from batch pretreatment with both LHW and EDA increased by 5–6% points when the temperature was increased from 140 to 180 °C. For flowthrough pretreatment, the higher temperature increased glucan content by 4% points. Figure 1 also shows that the flowthrough solids were 15–19% points richer in glucan than batch pretreated solids due to the low xylan and lignin content. On the other hand, the percent lignin in solids from batch pretreatment either remained at the 28% value of unpretreated poplar or increased to 30%. Overall, the distinctive feature in comparing the compositions of solids from flowthrough and batch pretreatments of poplar in Fig. 1 is the much lower lignin content of 18–20% for the former vs. 28–30% for the latter.

### Lignin removal

**Fig. 2:**
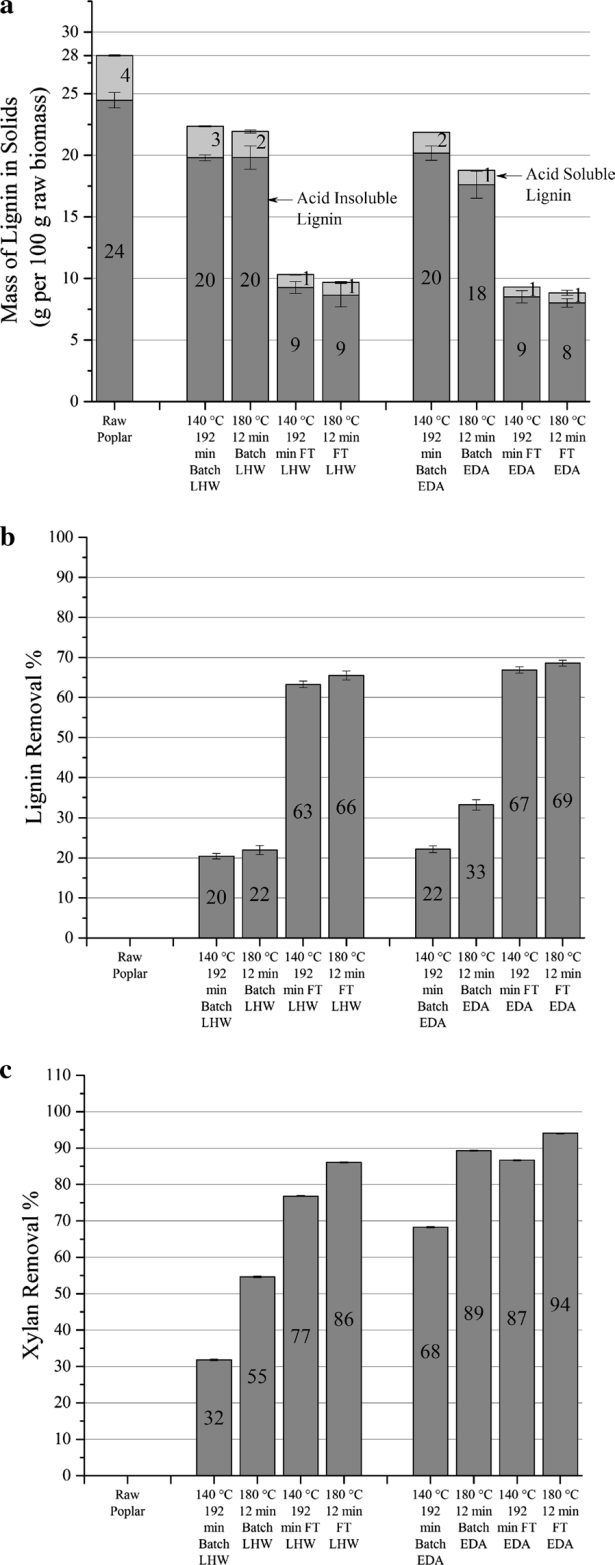
Mass of lignin, and lignin and xylan removal. Mass of lignin left in solids (**a**) and lignin (**b**) and xylan (**c**) removal from solids for batch and flowthrough (FT) pretreatments with just hot water (LHW) and extremely dilute acid (EDA)

As noted above, a distinctive characteristic of flowthrough pretreatment is lignin removal, as evidenced by Fig. 2 showing that flowthrough pretreatments significantly enhanced lignin removal to 63–69% compared to only 20–33% for batch pretreatments. For batch pretreatment at 140 °C, little difference was realized in lignin removal between LHW (20%) and EDA (22%), but increasing the temperature to 180 °C enhanced lignin removal to 33% for batch EDA operation while having little effect on lignin removal for LHW (22%). On the other hand, 180 °C LHW and EDA flowthrough pretreatments achieved lignin removals of 66 and 69%, respectively. Surprisingly, lower temperatures did not reduce lignin removals much in the flowthrough mode compared to 180 °C. At 140 °C for 192 min, lignin removals from flowthrough pretreatments were 63% in LHW and 67% in EDA. It is important to note that while lignin removal was only slightly enhanced in EDA compared to LHW flowthrough pretreatment, Zhang et al. [45] report a larger increase in lignin removal in EDA compared to LHW flowthrough pretreatment. This outcome can be due to their use of more severe pretreatment conditions ranging between 160 and 270 °C for 0–12 min at a linear velocity of 18.94 cm/min. This operation may have widened the gap between lignin removal in LHW and EDA flowthrough pretreatments, as a portion of lignin may deconstruct only at higher severity and/or temperature at acidic conditions.

Thus, out of 28 g of total lignin in unpretreated poplar, about 14–15 g was solubilized with flowthrough pretreatment at either temperature, as shown in Fig. 2a. By comparison, Liu and Wyman [27] found 65% lignin removal from corn stover at velocities of 2.8, 5.2, and 10.7 cm/min at 200 °C, and Archambault-Leger, Shao, and Lynd measured about the same lignin removal from poplar at 220 °C with LHW flowthrough pretreatment at a linear velocity of 8.66 cm/min and a 1.84 min residence time [33]. Thus, our results show that coupling a higher linear velocity of 15.8 cm/min with lower temperature operation was as effective in lignin removal as lower velocities combined with higher temperatures in these prior studies. Furthermore, the high velocities applied for flowthrough pretreatment likely prevented pseudo lignin deposition onto solids reported for batch pretreatments at these moderate severities [46, 47] and resulted in a substantial drop in lignin contents for the solids left after flowthrough pretreatment. Also interesting to note is that lignin was found to be largely soluble in the liquor just after flowthrough pretreatment and only a small amount of sediment was observed after storage of flowthrough pretreatment liquors at 4 °C. It was also observed that the 180 °C EDA flowthrough pretreatment liquor collected between 0 and 3 min was pink in color. This pink color was not seen during the liquor collected during heat-up to starting of pretreatment time (zero time). The pink color changed to light brown or yellow in liquor collection after 3 min till end of reaction. No other pretreatment condition presented this change of color as they all appeared light brown or yellow.

These results indicate that about 65–70% of the lignin being easier to deconstruct with pretreatment and subject to being removed if re-deposition is prevented. It is interesting to note that the more easily removed lignin fraction has a similar value to the fast reacting xylan fraction calculated for so many different biomass types. However, the coincidence may end there as most of the kinetically slower hemicellulose fraction can be solubilized at longer pretreatment times while the remaining lignin fraction has not been shown to be so amenable to deconstruction. Removal of a large fraction of the lignin by flowthrough pretreatment might be made possible by its bonding to far more soluble xylan. On the other hand, about 30–35% of the lignin may not be tightly associated with hemicellulose and, therefore, not swept from the system with hot water even when catalyzed by very dilute acid concentrations. This remaining fraction could be the most recalcitrant to deconstruction at aqueous conditions. This hypothesis is reinforced by about 65% of the lignin linkages in hardwood being β-*O*-4 bonds that are more easily broken than others [48] to form fragments subject to release by flowthrough pretreatment. It is worth noting that some of the results summarized above are for corn stover in which such linkages makeup about 60% of the total [49, 50]. Thus, the relatively easy breaking of some lignin–lignin bonds coupled with the high solubility of lignin-hemicellulose fragments and the ability of flowthrough pretreatment to remove these fragments before breaking of lignin-hemicellulose bonds allows less soluble lignin to condense back on the solids results in high removal of lignin by flowthrough pretreatment. On the other hand, if the lignin eventually breaks away from the hemicellulose and condenses onto the solids for batch systems, the extent of hemicellulose solubilization would be indicative of the degree of cycling of lignin from the solids into solution and back onto the solids in batch pretreatments, thereby disrupting lignin shielding of cellulose from enzyme action. In addition, although virtually 100% of hemicellulose can be removed by flowthrough and batch pretreatments with or without adding acid, these results point to about 30–35% of the lignin being highly recalcitrant to removal in aqueous environments.

Such a mechanism is able to explain lignin solubilization in hot water conditions that cannot be attributed to acid catalyzed cleavage of β-*O*-4 linkages in lignin due to an autohydrolysis reaction [21, 22]. As in LHW flowthrough pretreatment at relatively short residence times, acetic acid liberated from acetylated xylans is removed from the reactor before it can cause a drop in solution pH. It was found that at a flow rate of 20 mL/min through a 1 g biomass bed in the flowthrough reactor, acetic acid concentrations in the pretreatment liquors were so low that they could not be detected. Results from prior studies have supported the idea that xylan solubilization at elevated temperatures in hot water may not be solely due to acid catalysis due to liberation of organic acids (autohydrolysis) [26]. In addition, acetic acid has a low dissociation constant of 1.7 × 10^−5^ at 25 °C which drops to 0.6 × 10^−5^ at 150 °C [51]. In addition, softwood hemicelluloses are much less acetylated but still can show xylan yields similar to hardwood [28, 52]. This mechanism of solubilization of lignin-hemicellulose fragments also explains the linear relationship observed between lignin removal and xylan removal in flowthrough pretreatment [17].

### Xylan removal from the solids and yields in solution

**Fig. 3:**
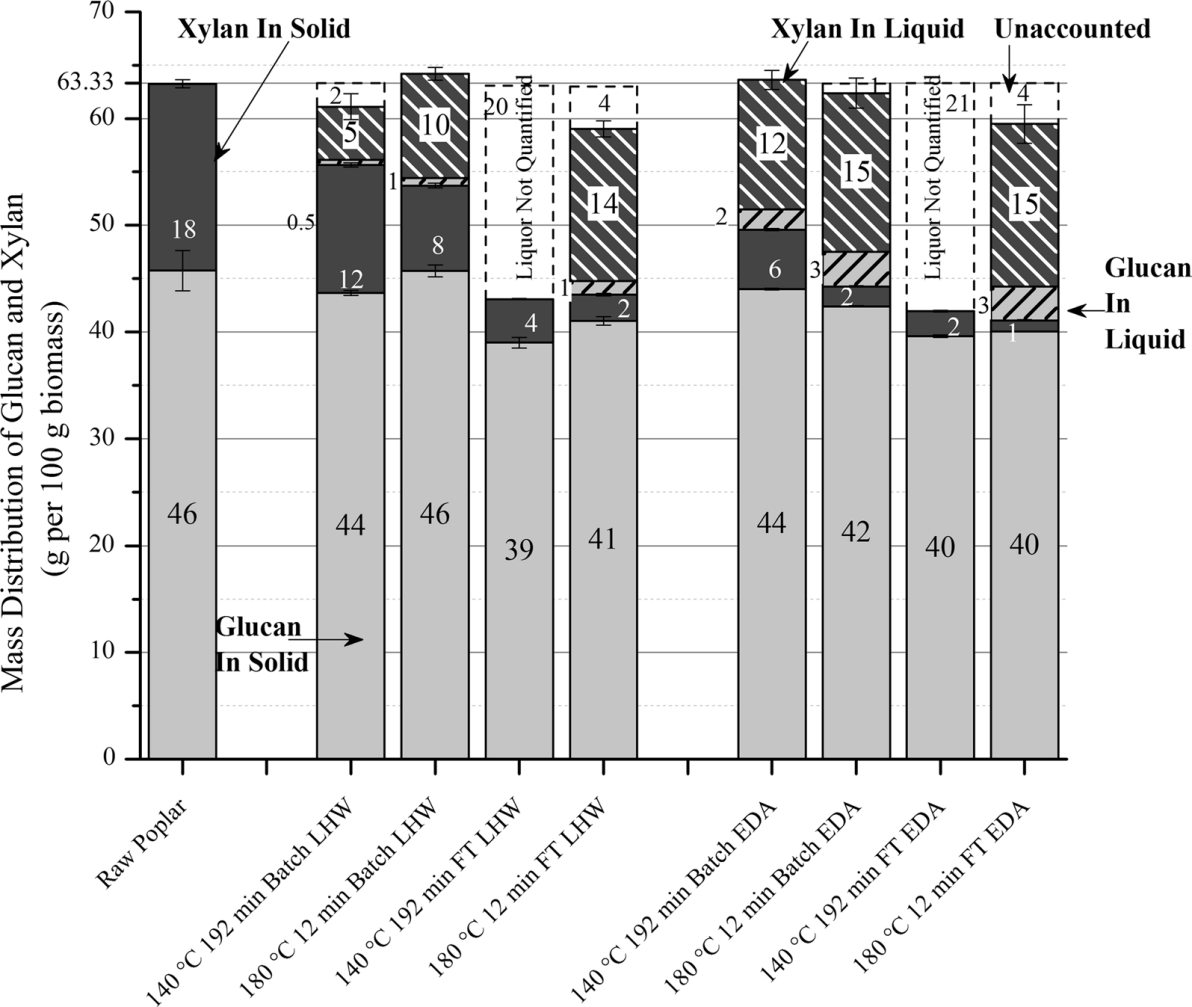
Mass distribution of glucan and xylan. Mass balance on glucan and xylan left in solids and solubilized in the liquid based on 100 g of initial poplar fed to pretreatment at selected temperatures and times for pretreatment with just hot water (LHW) and 0.05% sulfuric acid (EDA). Sugars could not be accurately measured for flowthrough pretreatment at 140 °C due to high volumes of water required for the long reaction times required. The “unaccounted” labels represent the inaccuracy in measurement of sugars in the pretreatment liquor. Number labels have been rounded-off to the nearest whole number for clarity

Consistent with the trends in Figs. 1, 2c and 3 show that mass of xylan solubilized increased with temperature and use of acid in both reactor configurations. This outcome is expected in that Saeman’s first order hemicellulose hydrolysis model predicts that the xylan solubilization rate increases with temperature and hydronium ion concentration [24, 25]. Overall, xylan removal ranged from 32 to 94%, with flowthrough pretreatment removing more than batch at otherwise equivalent conditions. Figure 3 shows LHW and EDA could remove about 5–14 g and 12–15 g xylan, respectively, out of 18 g in the unpretreated raw poplar. It is important to note that the mass of sugars in the liquors was not quantifiable for 140 °C flowthrough pretreatments as the liquid was too dilute (~10^−5^ g/L) for the extended time required for pretreatment. The performance for flowthrough pretreatment at 140 °C, therefore, had to be judged based on xylan removal from the solids. An (Additional file 1) shows material balances for all process conditions corresponding to the data in Fig. 3.

In Fig. 2c, the range of xylan removal was greater at LHW conditions (32–86%) than at EDA conditions (68–94%). However, xylan removal increased by about 34–36% points in going from LHW to EDA batch pretreatments for both temperatures run. For flowthrough pretreatments, the increase from LHW to EDA was only about 8–10% points. In either case, the increase in xylan removal from LHW to EDA was the same for both temperatures. Figure 2c also shows that LHW and EDA flowthrough pretreatments at 140 °C removed about 77 and 87%, respectively, of total xylan in raw biomass, while increasing the temperature to 180 °C increased xylan removal to 86 and 94% for LHW and EDA, respectively. Thus, flowthrough pretreatments removed large quantities of xylan even at 140 °C while batch pretreatment at 140 °C removed only 32% of the xylan for LHW and 68% xylan for EDA.

**Fig. 4:**
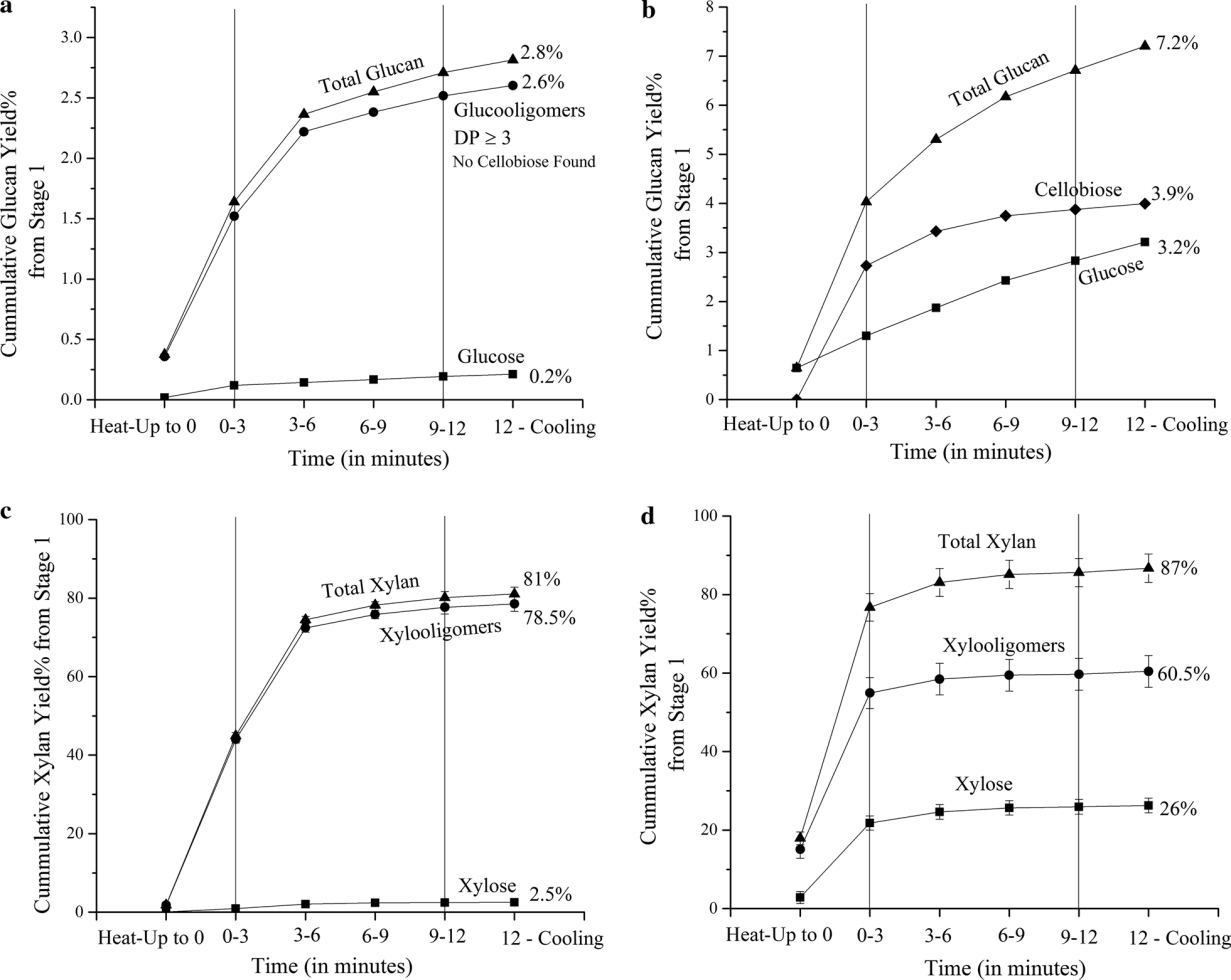
Glucan and xylan yields for flowthrough pretreatment at 180 °C. Yields of glucan recovered in solution as monomers, cellobiose, and higher degree of polymerization oligomers versus time for LHW (**a**) and EDA (**b**) pretreatments at 180 °C. Yields of xylan recovered in solution as monomers and xylooligomers versus time for LHW (**c**) and EDA (**d**) pretreatments at 180 °C

**Fig. 5:**
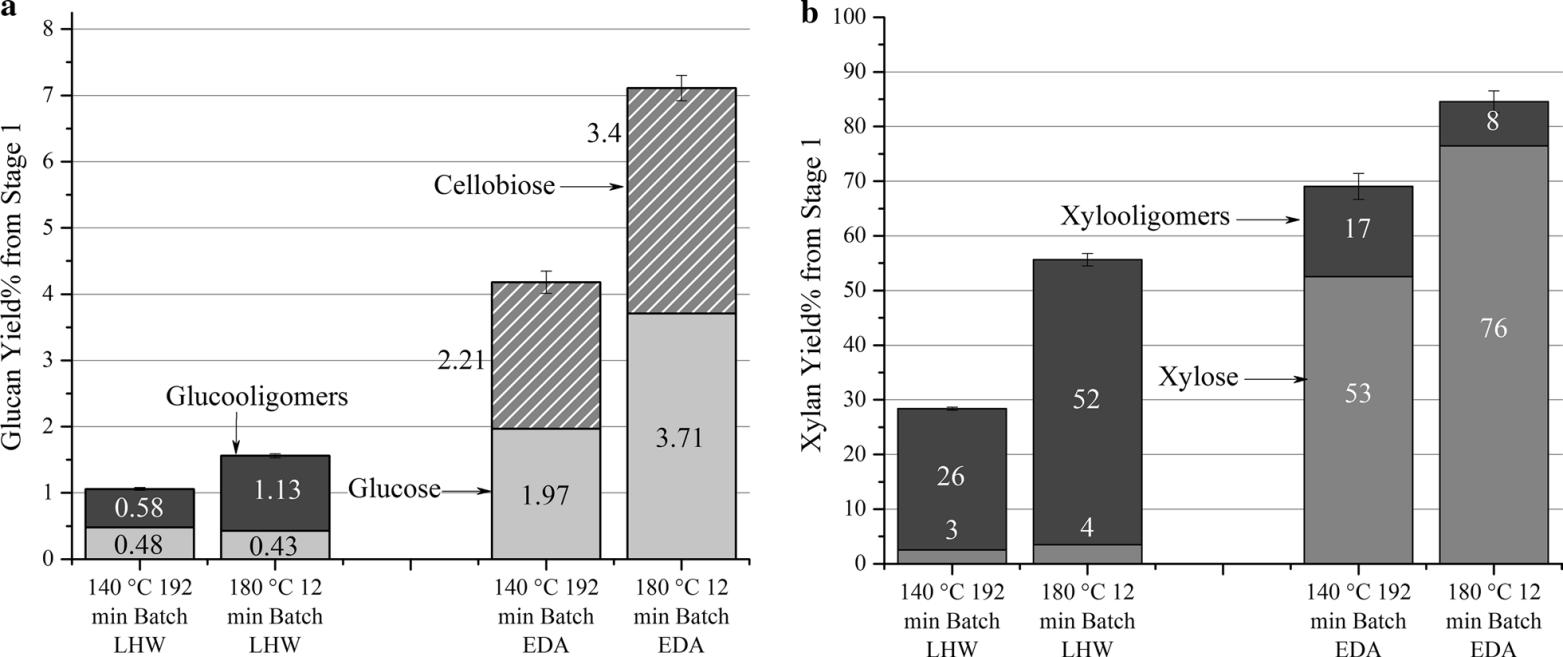
Glucan and xylan yields for batch pretreatment. Yields of glucose, cellobiose, and higher glucooligomers from batch LHW and EDA pretreatments at 140 and 180 °C (**a**) and of xylose and higher xylooligomers (**b**)

Figure 4 shows that LHW flowthrough pretreatment at 180 °C achieved an 81% xylan yield while Fig. 5 indicates that LHW batch operation at otherwise identical conditions only realized a xylan yield of 56%. However, EDA operation at 180 °C shrunk the difference in yields considerably in that batch operation increased substantially to 84% while in light of its already high yields from LHW operation, EDA flowthrough pretreatment could only enhance yields slightly relative to LHW operation to 87%. The smaller difference in xylan yield from flowthrough vs. batch EDA pretreatments at 180 °C is likely due to reaching a highly recalcitrant hemicellulose core that cannot be readily deconstructed. In either event, as noted in the past, first order homogenous kinetic models cannot explain why hemicellulose solubilization was so much greater for flowthrough than batch pretreatment at 180 °C [53]. Such models also cannot account for xylan removals from flowthrough pretreatment being so much closer for a 40 °C temperature difference than from batch pretreatment at the same severity factor. These results strongly suggest that mass transfer plays an additional role for xylan removal beyond what reaction kinetics alone can account for. It has been shown that a 10 mL/min flow rate to a flowthrough reactor increased mass transfer coefficients by 90% compared to those for stirred batch pretreatment of corn stover at 180 °C [26]. Brennan and Wyman [54] developed two mass transfer models to explain hemicellulose hydrolysis, with one attributing the difference to shrinking the liquid film thickness. The other biphasic leaching model suggested that by continually preventing equilibrium between xylan in the biomass solids and its concentration in the pretreatment liquor, xylan removal is solubilized until exhaustion. Another branched pore leaching model, developed from leaching of water soluble organic carbon from soil [55], was able to describe hemicellulose hydrolysis based on mass transfer alone.

The 86% xylan removal at 180 °C for 12 min of LHW flowthrough pretreatment was higher than the 62% value reported for operation at 180 °C for 8 min and 80% at 200 °C for 16 min by Archambault-Leger, Shao, and Lynd [56] for poplar. However, this difference could be due to the fact that they applied a flow rate of 30 mL/min in their 2.1 cm internal diameter flowthrough reactor to produce a linear velocity of about 8.66 cm/min and a residence time of about 1.85 min compared with our velocity of 15.8 cm/min and residence time of 0.96 min. Mok and Antal [18] reported 100% hemicellulose removal from solids and about 90% hemicellulose sugar recovery for LHW flowthrough pretreatment of 10 biomass species at 230 °C for 2 min. They pointed out that their results were not strongly dependent on temperature or time within the range of 200 to 230 °C for 0–15 min at a flow rate of 1 mL/min. Liu and Wyman’s study [27] of LHW flowthrough pretreatment of corn stover at 200 °C showed that increasing the linear velocity from 2.8 to 10.7 cm/min increased the rate of biomass solubilization and xylose recovery, although both parameters eventually reached the same values after 16 min.

Figure 5 shows that only 3–4% of the solubilized xylan was monomeric xylose with all the rest being oligomers for LHW batch pretreatments at both temperatures. However, addition of a small amount of acid increased the percent monomeric xylose to 53% at 140 °C and 76% at 180 °C. Production of monomers is advantageous in that they are more universally suitable for yeast uptake to produce ethanol while oligomers are not [57]. The small amount of acid also enhanced overall xylan solubilization in addition to converting more of the xylooligomers to xylose.

Figure 4c, d show liquor profiles versus time of LHW and EDA, respectively, flowthrough pretreatment at 180 °C. Xylooligomers were predominant for 180 °C LHW flowthrough pretreatment with limited monomeric xylose (2.5%). Although we were unable to characterize the extremely dilute liquor for 140 °C LHW flowthrough pretreatment, most of the xylan is expected to be oligomeric for those conditions as well. On the other hand, 180 °C EDA flowthrough pretreatment produced about 30% of the xylan in solution as monomers (26% out of total 87% xylan). Previous characterization of the degree of polymerization (DP) of xylooligomers produced by LHW flowthrough pretreatment of corn stover at 200 °C for 10 min showed that high linear velocities such as 19.7 cm/min (25 mL/min) resulted in DPs greater than 30 for most of the xylooligomers while longer residence times and lower flow rates or higher temperatures reduced the xylooligomer DP [58].

The fast and slow reacting hemicellulose model introduced by Kobayashi and Sakai [25] for dilute acid conditions and improved by others to include sugar oligomer intermediates could help understand these patterns in xylose and xylooligomer yields from LHW and EDA flowthrough pretreatments. Past fits of this model to data attributed about 65% of the reacted xylan to be fast reacting for many biomass types [59], and Shen and Wyman [60] showed that modeling the fast reaction to oligomers as a reversible first order reaction improved the data fit. For the latter, the fast reacting xylan fraction can either form xylooligomers reversibly or react directly to xylose, while the slow reacting xylan forms xylooligomers that breakdown to xylose that eventually decompose into furfural. The model showed that the slowest relative step for 180 °C LHW pretreatment was xylooligomer conversion to xylose, while the slowest relative step for 160 °C dilute acid pretreatment was xylose conversion to furfural. In addition, the rate constant for dilute acid breakdown of xylooligomers to xylose was about 100 times greater than for LHW. They also showed that the rate constant for direct xylan conversion to xylose was about 5 times greater for dilute acid than LHW. Thus, these and other insights gained by this model predict patterns similar to those observed in this study of high amounts of oligomers for LHW, more monomers for EDA and enhanced yields from EDA pretreatment. Furthermore, the release of 20% of the total initial xylan as xylose and 55% as xylooligomers in the first 3 min from EDA flowthrough pretreatment at 180 °C shown in Fig. 4 is consistent with conclusions by a number of studies that about 65–70% of the xylan is more easily hydrolyzed while the rest would take a longer time to appear in solution. Furthermore, xylan side chains may affect solubilization, e.g., reducing methylation of glucuronic acid side chains of xylan in *Arabidopsis* led to increased solubilization with LHW pretreatment [61].

### Glucan removal from the solids and yields in solution

The material balances presented in Fig. 3 reveal that very little glucan was solubilized by any of the batch pretreatments, resulting in the dissolved glucan yields in Fig. 5 for LHW being only about 1 and 1.5% at 140 °C and 180 °C, respectively, while those for EDA increased only slightly to about 4 and 7% for the same temperature order. The liquor from LHW batch pretreatment contained both glucose and glucooligomers other than cellobiose, but only glucose and cellobiose were measurable in the liquid from batch EDA operations. For 180 °C flowthrough pretreatment, the LHW yields presented in Fig. 4 were 0.2% for glucose and 2.6% for glucooligomers with no cellobiose, while only glucose and cellobiose were observable in the EDA flowthrough pretreatment liquor. Total glucan yields were both about 7% for EDA batch and EDA flowthrough pretreatment at 180 °C, with the yields continually increasing with time of flowthrough pretreatment. A possible source for the 2–4% monomeric xylose yields and less than 2% glucan yields from LHW pretreatments could be the loose structures of xyloglucans and amorphous cellulose in the primary cell walls [62]. Perhaps small amounts of glucose were also derived from cellulose in the secondary cell walls. The patterns of glucose release have similarities to predictions by fast reacting xylan models in that (1) almost all of glucan and xylan from LHW pretreatment is in oligomeric form, (2) glucose and xylose are released right from the very start for 180 °C EDA flowthrough pretreatment, and (3) glucan yields were enhanced by about 5% for both batch and flowthrough EDA pretreatments.

### Enzymatic hydrolysis

**Fig. 6:**
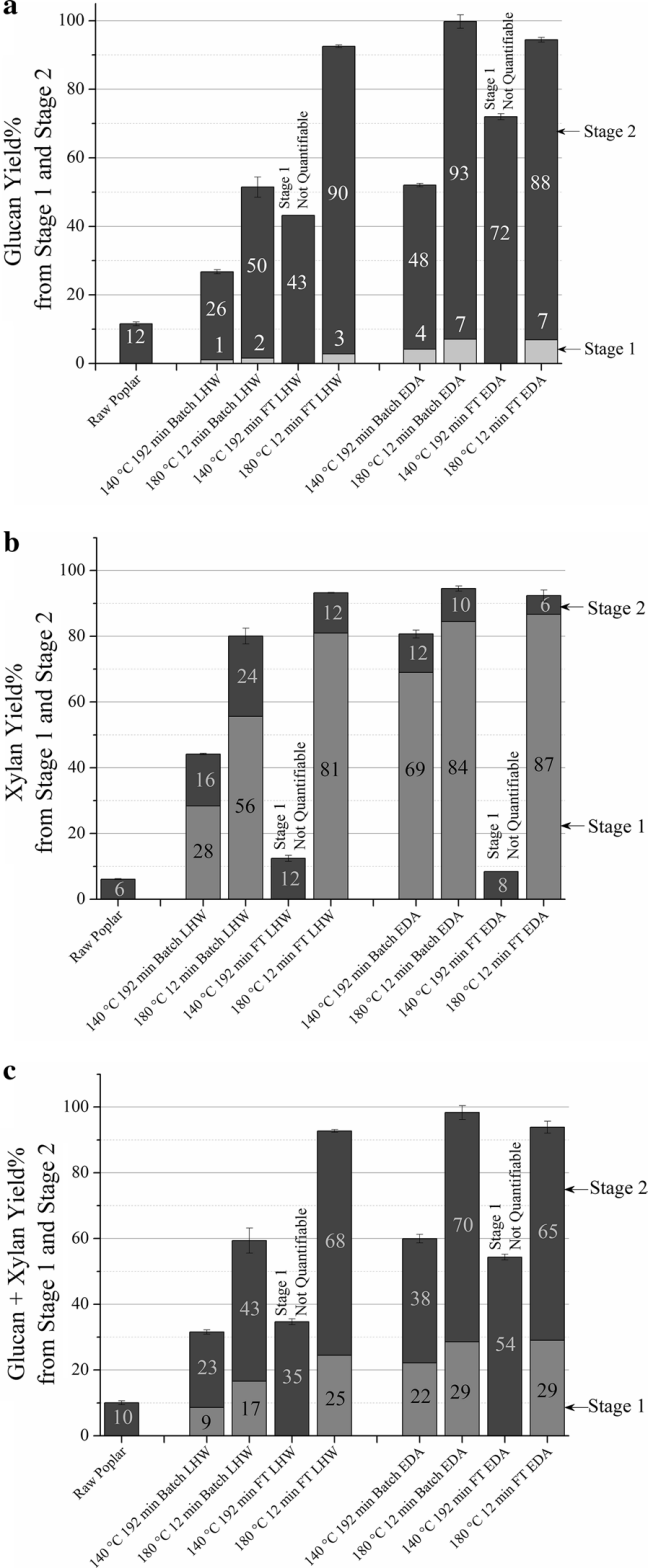
Stage 1 and Stage 2 sugar yields. Glucan (**a**), xylan (**b**), and glucan + xylan (**c**) yields from Stage 1 and Stage 2 combined. Stage 2 enzymatic hydrolysis was performed with 100 mg cellulase/g glucan initially in the raw poplar using flasks shaken at 150 rpm for 120 h at 50 °C

Figure 6 shows yields of glucan, xylan, and glucan plus xylan recovered in solution from both LHW and EDA pretreatments (Stage 1) combined with subsequent enzymatic hydrolysis (Stage 2) of the pretreated solids. A high enzyme loading of 100 mg protein/g glucan in unpretreated biomass was applied to the pretreated solids as our focus was on understanding substrate features that contribute to recalcitrance and avoiding being distracted by enzyme limitations due to such factors as enzyme inhibition or loss of activity. Without any pretreatment, even at this high enzyme loading, Stage 1 + 2 glucan + xylan yields (total sugar yield) was 10% at best. On the other hand, among the four batch pretreatment conditions applied, EDA at 180 °C stood out in achieving near theoretical glucan yield (99%). One reason for this superior performance could be that the 33% lignin removal for this condition was about 11–13% higher than that for the other three batch pretreatments. Raising the temperature from 140 to 180 °C had a greater effect on increasing total glucan yields for EDA batch pretreatments (52 vs. 100%) than for LHW batch pretreatments (27 vs. 52%). Among flowthrough pretreatments, 93 and 94% sugar deconstruction could be achieved at 180 °C for LHW and EDA conditions, respectively. A small quantity of sugar could not be accounted for during flowthrough pretreatments, possibly due to inaccuracy in measurement of low concentrations of glucose in liquors or minor loss of pretreatment liquor during the collection with multiple flasks for sugar vs. time curves. This is why Stage 1 sugar yields from 180 °C flowthrough pretreatments seem to be slightly lower than 180 °C EDA batch pretreatment. However, considering the pretreated solid composition data in Fig. 1 and mass balances in Fig. 3, both for 180 °C flowthrough pretreatments, led to near theoretical Stage 1 + 2 glucan plus xylan yields, similar to 180 °C EDA batch pretreatment (99%). Now, in spite of high lignin removal, Stage 2 glucan + xylan yields produced by 140 °C LHW and EDA flowthrough pretreatments were 35 and 54% compared to 68 and 65% at 180 °C, respectively. Thus, lignin removal by flowthrough pretreatment or lignin relocation by batch pretreatment at lower temperature is not sufficient to realize high digestion by enzymes. The results from this study point to the likelihood that the more recalcitrant 30–35% of the lignin that could not be removed by flowthrough pretreatment or relocated by batch operation is likely a primary contributor to biomass recalcitrance which require higher temperatures to affect such lignin properties as surface spread on cellulose, strength of association with cellulose, and hydrophobicity. However, other factors could play a role such as the overall cellulose surface area that is available for adsorption by the carbohydrate binding module (CBM) of fungal cellulases, specifically the hydrophobic face of cellulose crystals [63], might be affected by differences in pretreatment pH and temperature.

## Conclusions

The results from this study suggests that about 30–35% of the lignin in poplar that could not be removed by flowthrough pretreatment with or without adding acid is an important contributor to the recalcitrance of biomass to enzymatic digestion. These results also point to the possibility that hemicellulose removal is more important as an indicator of lignin disruption than in playing a direct role in reducing biomass recalcitrance. Moreover, solids from 180 °C flowthrough pretreatment were much more digestible than solids pretreated at 140 °C despite similar lignin and extensive hemicellulose removal for both, suggesting that higher temperatures play a vital role in improving cellulose digestibility through modifying lignin and/or other barriers. Adding a small amount of acid disproportionately enhanced hemicellulose solubilization for both batch and flowthrough pretreatments. Use of extremely dilute acid in batch pretreatment led to high recovery of xylan in the form of monomeric xylose. Flowthrough pretreatment conditions applied here were used to study pretreatment mechanisms and led to very dilute sugar concentrations. Conditions that result in high product concentration are needed for industrial applications of flowthrough pretreatment technology.

## Additional file

**Additional file 1.** Detailed material balances for Stage 1 (pretreatment) and Stage 2 (enzymatic hydrolysis) for all process conditions.

## Abbreviations

LHW: liquid hot water
EDA: extremely dilute acid
BESC: Bioenergy Science Center
BESC STD: BESC standard poplar
