# Supplementary material for: Flowthrough pretreatment with very dilute acid provides insights into high lignin contribution to biomass recalcitrance
Source: Biotechnol Biofuels. 2016 Nov 10;9:245. doi: 10.1186/s13068-016-0660-5 (PMC5103384; doi:10.1186/s13068-016-0660-5)
Supplement: Supplementary file 1 — Additional file 1. Detailed material balances for Stage 1 (pretreatment) and Stage 2 (enzymatic hydrolysis) for all process conditions. [file 13068_2016_660_MOESM1_ESM.pdf]

Flowthrough pretreatment with very dilute acid provides insights into high lignin contribution to biomass recalcitrance

Material Balances

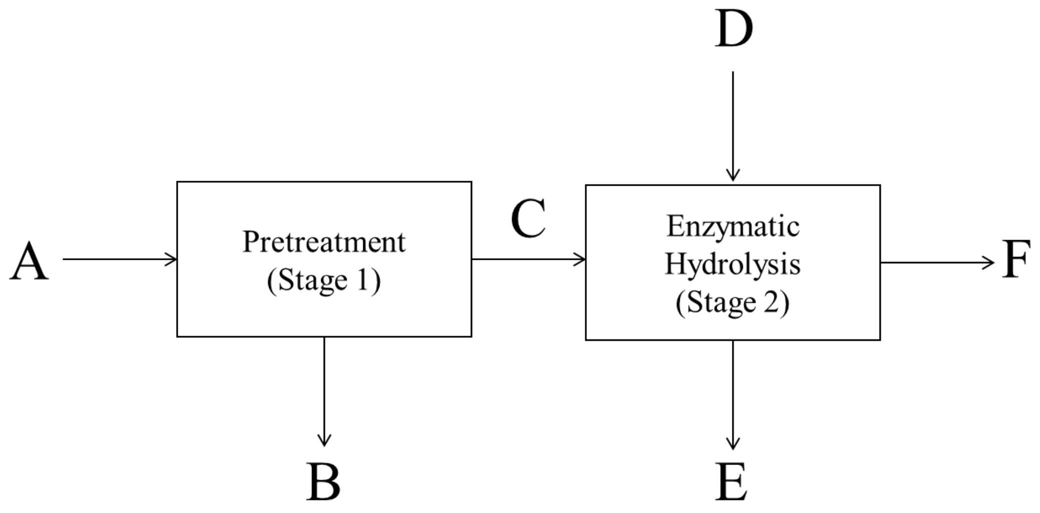

100 kg biomass basis

Enzyme after reaction assumed to remain in biomass residue.

Table 1. Materials in Stream A (in kilograms)

Condition	Mass of Solid	Glucan in Raw	Xylan in Raw	Lignin in Raw	Others in Raw	Acid to Stage 1	Water To Stage 1
No pretreatment	100	45.75	17.57	28.10	8.58	0	0
140°C 192 min Batch LHW	100	45.75	17.57	28.10	8.58	0	4900
180°C 12 min Batch LHW	100	45.75	17.57	28.10	8.58	0	4900
140°C 192 min FT LHW	100	45.75	17.57	28.10	8.58	0	396000
180°C 12 min FT LHW	100	45.75	17.57	28.10	8.58	0	36000
140°C 192 min Batch EDA	100	45.75	17.57	28.10	8.58	2.45	4898
180°C 12 min Batch EDA	100	45.75	17.57	28.10	8.58	2.45	4898
140°C 192 min FT EDA	100	45.75	17.57	28.10	8.58	198	395802
180°C 12 min FT EDA	100	45.75	17.57	28.10	8.58	18	35982

Table 2. Materials in stream B (in kilograms)

Condition	Total Mass of Liquid	Glucose in Pretreatment Liquor	Cellobiose in Pretreatment Liquor	Glucooligomers in Pretreatment Liquor	Xylose in Pretreatment Liquor	Xylooligomers in Pretreatment Liquor	Solublizied Lignin in Pretreatment Liquor	Others in Pretreatment Liquor
No pretreatment	0	0.00	0.00	0.00	0.00	0.00	0.00	0.00
140°C 192 min Batch LHW	4919	0.22	0.00	0.27	0.45	4.54	5.75	5.11
180°C 12 min Batch LHW	4924	0.20	0.00	0.52	0.62	9.16	6.18	7.39
140°C 192 min FT LHW	396047	N/A	N/A	N/A	N/A	N/A	17.78	8.58
180°C 12 min FT LHW	36047	0.10	0.00	1.19	0.44	13.80	18.42	7.73
140°C 192 min Batch EDA	4927	0.90	1.01	0.00	9.23	2.90	6.24	7.13
180°C 12 min Batch EDA	4936	1.70	1.56	0.00	13.43	1.42	9.33	6.97
140°C 192 min FT EDA	396049	N/A	N/A	N/A	N/A	N/A	18.79	8.58
180°C 12 min FT EDA	36050	1.47	1.83	0.00	4.61	10.62	19.27	8.58

Table 3. Materials in stream C (in kilograms)

Condition	Mass of Solid (dry basis)	Glucan in Pretreated Solid	Xylan in Pretreated Solid	Lignin in Pretreated Solid	Others in Pretreated Solid
No pretreatment	100	45.75	17.58	28.10	8.57
140°C 192 min Batch LHW	81	43.66	11.98	22.36	2.89
180°C 12 min Batch LHW	76	45.74	7.97	21.93	0.61
140°C 192 min FT LHW	53	38.52	4.08	10.32	0.00
180°C 12 min FT LHW	53	41.05	2.45	9.69	0.27
140°C 192 min Batch EDA	73	44.99	5.58	21.87	0.87
180°C 12 min Batch EDA	64	42.39	1.88	18.77	1.03
140°C 192 min FT EDA	51	39.61	2.34	9.31	0.00
180°C 12 min FT EDA	50	40.05	1.04	8.83	0.00

Table 4. Materials in stream D (in kilograms)

Condition	Enzyme to Stage 2	Water to Stage 2	Citrate Buffer to Stage 2	Antibiotic to Stage 2	Total Reaction Mass of EH
No pretreatment	4.58	4410	59.03	0.92	4575
140°C 192 min Batch LHW	4.58	4223	56.33	0.87	4366
180°C 12 min Batch LHW	4.58	4433	59.02	0.91	4574
140°C 192 min FT LHW	4.58	3744	49.70	0.77	3852
180°C 12 min FT LHW	4.58	3993	52.96	0.82	4105
140°C 192 min Batch EDA	4.58	4362	58.05	0.90	4499
180°C 12 min Batch EDA	4.58	4115	54.70	0.85	4239
140°C 192 min FT EDA	4.58	3853	51.10	0.79	3961
180°C 12 min FT EDA	4.58	3898	51.67	0.80	4005

Table 5. Materials in stream E (in kilograms)

Condition	Total Mass of EH Liquor	Glucan in EH liquor	Xylan in EH Liquor	Buffer in EH Liquor	Antibiotic in EH Liquor
No pretreatment	4477	5.28	1.07	59.03	0.92
140°C 192 min Batch LHW	4295	11.75	2.78	56.33	0.87
180°C 12 min Batch LHW	4520	22.82	4.30	59.02	0.91
140°C 192 min FT LHW	3816	19.78	2.19	49.70	0.77
180°C 12 min FT LHW	4090	41.05	2.16	52.96	0.82
140°C 192 min Batch EDA	4445	21.89	2.06	58.05	0.90
180°C 12 min Batch EDA	4215	42.39	1.77	54.70	0.85
140°C 192 min FT EDA	3940	32.93	1.48	51.10	0.79
180°C 12 min FT EDA	3992	40.05	1.00	51.67	0.80

Table 6. Materials in stream F (in kilograms)

Condition	Total Mass of Residue	Glucan in Residue	Xylan in Residue	Lignin in Residue	Others in Residue	Enzyme in Residue
No pretreatment	98	40.47	16.51	28.10	8.57	4.58
140°C 192 min Batch LHW	71	31.90	9.20	22.36	2.89	4.58
180°C 12 min Batch LHW	54	22.92	3.67	21.93	0.61	4.58
140°C 192 min FT LHW	35	18.74	1.89	10.32	0.00	4.58
180°C 12 min FT LHW	15	0.00	0.29	9.69	0.27	4.58
140°C 192 min Batch EDA	54	23.10	3.52	21.87	0.87	4.58
180°C 12 min Batch EDA	24	0.00	0.11	18.77	1.03	4.58
140°C 192 min FT EDA	21	6.67	0.87	9.31	0.00	4.58
180°C 12 min FT EDA	13	0.00	0.04	8.83	0.00	4.58
